# Postauricular injection glucocorticoid in the treatment of sudden hearing loss: A protocol for systematic review and meta-analysis

**DOI:** 10.1097/MD.0000000000023241

**Published:** 2020-11-20

**Authors:** Jiao Liang, Hui Xie, Han-Jen Chiang, Sha Li, Zhiqing Liu, Jiongke Li, Chenyi Zeng

**Affiliations:** Hospital of Chengdu University of Traditional Chinese Medicine, No. 39 Shi-er-qiao Road, Chengdu, Sichuan Province, PR China.

**Keywords:** meta-analysis, postauricular injection, sudden hearing loss, systematic review

## Abstract

**Background:**

Sudden hearing loss is an emergency health problem in the Department of Otolaryngology that must be treated in a timely manner, or may cause lifelong regrets. The application of postauricular injection of glucocorticoid is a popular treatment to recover patients hearing level in recent years. However, the effectiveness and safety of postauricular injection of glucocorticoid needs to be assessed systematically.

**Methods and analysis:**

The purpose of the study is to undertake a systematic review and meta-analysis on the effectiveness and safety of postauricular injection of glucocorticoid to treat patient diagnosed with sudden hearing loss. We will search the following databases from the date of publication to July 1, 2020: PubMed, EMBASE, Web of Science, the Cochrane Library, CNKI, Wanfang databases, the Chinese Biomedical Literature Database (CBM), the Chinese Science and Technology Periodical Database (VIP) and the Chinese Cochrane Centre's Clinical Trial Registry Platform. Observational studies regarding the association between postauricular injection of glucocorticoid and sudden hearing loss were written in English and Chinese were included. RevManV.5.3 software will be used for meta-analysis. According to the heterogeneity of the research results, fixed effects model, random effects model, subgroup analysis, sensitivity analysis, and others will be used. Ethics approval was not required for this protocol. The findings will be disseminated through journal articles and conference presentations.

**Results:**

Objectively, evaluate the efficacy and safety of postauricular injection of glucocorticoid for sudden hearing loss.

**Conclusion:**

To provide evidence-based medicine for glucocorticoid treatment methods in patients with sudden hearing loss.

**OSF registration number:**

DOI 10.17605/OSF.IO/N5RV3.

## 1 Introduction

Sudden hearing loss (also known as sudden deafness) refers to a sensorineural hearing loss that occurs suddenly in 1 or both ears within 72 hours; presented ≥30 dB at 3 consecutive frequencies, and the cause is unknown.^[1]^ The incidence of sudden deafness has increased year by year. In the past, the disease was thought to be self-healing and no special treatment was required.^[2]^ However, there is an increase in number of patients in recent years seeking treatment for this disease, the awareness of the consequences of this disease has gradually increased.^[3]^ Comprehensively, experts have reached a consensus that this disease is a common emergency in the Department of Otolaryngology and needs to be treated in a timely manner.^[1,2]^ If the best treatment time has been missed, it may leave lifelong regrets for the patient.

Since the guideline for sudden deafness appear, glucocorticoids have been one of its effective treatments.^[1,4,5]^ In 2019, the updated version of the U.S. clinical practice guidelines for sudden deafness glucocorticoids is still the recommended drug.^[1,6]^ The drug can be used in systemic or topical ways, but scholars in China and abroad have disputes about its uses.^[7,8]^ Postauricular injection is listed as a type of topical medication. In recent years, many experts have done a lot of research on the treatment of sudden deafness by injecting glucocorticoid behind the ear; injecting glucocorticoid around the stria vascularis, the cochlea, and the lymphatic vessels.^[5,9,10]^ There is no difference in the distribution at the superior and inferior of the cochlea.^[11,12]^ The blood labyrinth barrier and drug penetration have led to insufficient effective concentration of drugs reaching the inner ear when used in systemic ways. The blood labyrinth barrier can be avoided by injection behind the ear, so the drug concentration in the local and inner ear tissues is higher than the systemic drug, the duration is longer, the local bioavailability is higher, and the drug concentration in the Cortidevice is higher.^[5,13]^

At present, some experts have proposed that the efficacy and safety of postauricular injection are better than tympanic injection and systemic medication, and have conducted a clinical randomized controlled study on this.^[14,15]^ Therefore, a systematic evaluation and meta-analysis of the effectiveness and safety of glucocorticoid injection behind the ear is necessary, and it is expected to provide a reference for clinical selection.

## 2 Research method

### 2.1 Registration details and ethics

This work has been registered on the OSF. The registration number is DOI 10.17605/OSF.IO/N5RV3. The study is conducted with the requirements of the reporting rules in the (PRISMA-P) (Preferred Reporting Items for Systematic Reviews and Meta-Analyses Protocols),^[17]^ and this work is a systematic review, the data used in this systematic review are all from the published literature, so there is no need to submit it to the ethics committee for review.

### 2.2 Included and excluded criteria

#### 2.2.1 Study design types

Randomized controlled trial (RCT) of glucocorticoid injection behind the ear to treat SHL is chosen as study design. The grey literature database, conference literature database, and completed or ongoing trial data from various clinical trial registration databases will be include in our study.

#### 2.2.2 Participants

Participants for the study would be chosen based on the Sudden Hearing Loss Clinical Practice Guidelines by United States issued in 2012^[1]^ and the Guidelines for the Diagnosis and Treatment of Sudden Hearing Loss in 2015^[18]^ formulated by experts in China. Diagnostic criteria for SHL include:

1. Sudden hearing loss is defined as a rapid onset, occurring over a 72-hour period, of a subjective sensation of hearing impairment in one or both ears,
2. the PTA test show decrease in hearing of ≥30 decibels (dB), affecting at least 3 consecutive frequencies.

Besides, in this study we have to rule out stroke, nasopharyngeal carcinoma, acoustic neuroma and other serious diseases. Regardless of patients high blood pressure, diabetes, race, age, gender, and the duration of the disease.

#### 2.2.3 Interventions

Including the RCT trials of glucocorticoids for SHL, regardless of the type of SHL, dosage and manufacturer of glucocorticoids, frequency of use and duration of treatment with glucocorticoids. In the experimental group, glucocorticoids were injected behind the ear, the control group was given other methods of glucocorticoids (systemic medication such as oral or intravenous infusion, local medication such as intratympanic injection), and same for other interventions, and they could be given simultaneously or could be given with or without conventional treatment (drugs like thrombolytics, vasodilators, vasoactive sub-stances, or antioxidants and hyperbaric oxygen). The control group will not include other clinical treatment methods for SHL, such a, aromatherapy, acupuncture, moxibustion, yoga, traditional Chinese exercises, and Chinese herbs. The following types of interventions will be included.

1. Postauricular injection glucocorticoid VS conventional treatment
2. Postauricular injection glucocorticoid+ conventional treatment VS Intravenous glucocorticoid + conventional treatment
3. Postauricular injection glucocorticoid+ conventional treatment VS Oral glucocorticoid + conventional treatment
4. Postauricular injection glucocorticoid+ conventional treatment VS Intratympanic glucocorticoid + conventional treatment
5. Postauricular injection glucocorticoid VS no treatment
6. Postauricular injection glucocorticoid VS Intravenous glucocorticoid
7. Postauricular injection glucocorticoid VS Oral glucocorticoid
8. Postauricular injection glucocorticoid VS Intratympanic glucocorticoid

### 2.3 Outcome measures

#### 2.3.1 Primary outcome

The primary outcome will be chosen by clinical efficacy. The curative effect standards for sudden deafness are the clinical practice guidelines by American published in 2012 and the Guidelines for the diagnosis and treatment of sudden hearing loss in 2015 by Chinese experts.^[1,20]^

#### 2.3.2 Secondary outcome

Length of hospital stay, adverse reactions and adverse events will be included

### 2.4 Literature resources

To obtain as comprehensive clinical research data as possible we will search literature database and other clinical trial registration platforms like Chinese Clinical Trial Register, UK National Research Register, UK Clinical Trial Gateway, and WHO ICTRP and so on.

#### 2.4.1 Literature database search

Electronic retrieval of all documents from the establishment of the database to July 2020. The retrieval database includes PubMed, EMBASE, Web of Science, the Cochrane Library, CNKI, Wanfang databases, the Chinese Biomedical Literature Database (CBM), the Chinese Science and Technology Periodical Database (VIP) and the Chinese Cochrane Centre's Clinical Trial Registry Platform. The retrieved literature language is limited to Chinese or English.

We will use subject words and free words to search every database. The search formula is follows: (“Sudden Hearing Loss” OR “Deafness, Sudden” OR “Sudden Deafness” OR “Sudden Sensorineural Hearing Loss” OR “Hearing Loss”) AND (“Postauricular injection” OR “Injection behind Ear”) AND (“randomized clinical trial” OR “randomized controlled trial”). The search operators and search fields will be accurately converted to the search strategies of other databases.

### 2.5 Literature screening

First, import all the found references into EndNote X9 software, and perform grouping and duplicate document checking.

**Figure 1:**
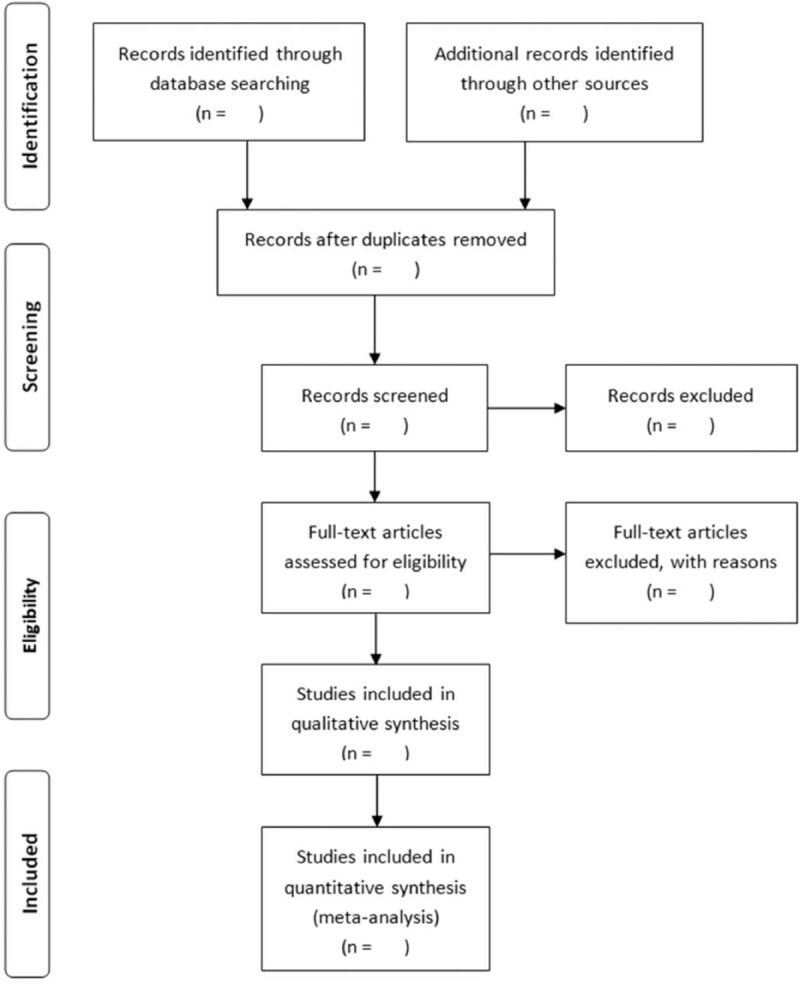
Flow diagram of the identification and selection studies.

Two authors (LS, CHJ) who have received evidence-based medicine training will screen all documents according to the inclusion and exclusion criteria, and eliminate repetitive documents based on authors, journals and abstracts, page numbers, etc., and finally form a new folder. The reviewer (LJ) obtains the full text of the article in the new folder, and analyzes again strictly against the inclusion criteria, and details the reasons for the excluded documents And recorded in excel, if there is a dispute in the process, it will be resolved through discussion or a third party (XH) review and judgment. The specific process is shown in the Figure 1.

### 2.6 Data extraction

Read the full text that meets the conditions, and the 2 authors (LS, CHJ) will extract the original data according to the document extraction table. If there is a dispute during the extraction process, first discuss it. If the dispute still exists, the author (XH) will make a decision judgment. For missing records in the included literature, we will contact the authors via email and try our best to improve relevant information. The structure of the document extraction table is as follows:

1. Basic information: number of literature, title of literature, first author, country and region, years of publication, type of funding, name of journal, language of language, ethical approval or not, conflict of interest or not.
2. Research methods: research design type, sample size, randomization method, random quality, between groups, blind method, completeness of research data, statistical methods.
3. Patient information: baseline level, course of disease, diagnostic criteria, efficacy criteria.
4. Intervention information: intervention type, intervention time, intervention frequency, intervention result.
5. Result information: main/minor result value, measurement method, measurement time, adverse reaction, adverse event.

### 2.7 Data analysis and synthesis

Using RevMan5.3 software to test quantitative synthesis and analysis of data. When *P * >* *.01, *I*^2^* *≤* *50%, using the fixed effect model to analysis; when *P *≤* *.01, *I*^2^ > 50%,using the random effect model, but the results should be carefully explained; when *P *≤* *.01, *I*^2^ > 75%, descriptive analysis will be used.

#### 2.7.1 Assessment of risk of bias

Two statistically-trained authors (LZQ and LS) conducted independent risk assessments on relevant literature according to the Cochrane Review Handbook 5.1.0.^[19]^ If there is any inconsistency, discuss it first, and if there is no agreement, it will be handed over to the third author (XH). Ruling the risk of bias mainly includes (random method, allocation concealment, blind method, withdrawal or loss to follow-up). There are 3 levels of risk of bias: high, low and unclear. The evaluation results are generated through Review Manager 5.3 software to produce specific graphics. If the risk of bias is unclear, and we will try our best to contact the author via email to obtain this information.

#### 2.7.2 Publication bias

If the number of clinical trials is ≥10, we will use Egger to check the funnel plots symmetry.

#### 2.7.3 Subgroup analysis

Subgroup analysis will be used if the included studies have some heterogeneity.

#### 2.7.4 Sensitivity analysis

We will use sensitivity analysis to evaluate the reliability of the meta-analysis results. If excluding a certain document will lead to significant changes in sensitivity before and after, a careful analysis of relevant literature is required.

## 3 Discussion

With the increase of life pressure, the incidence of sudden deafness is also increasing. Although the treatment of sudden deafness is constantly changing, the use of glucocorticoids is still the recommended drug, how to avoid the side effects of glucocorticoid drugs, such as, Insomnia, tympanic membrane perforation, insufficient drug concentration, etc., the use of topical medication is becoming to attention by clinicians.^[16]^

The method of injection behind the ear not only can avoid the side effects of systemic glucocorticoid drugs, but also, can be applied to people who are contraindicated with glucocorticoids, such as diabetes and gastric ulcer patients. Therefore, the injection of glucocorticoids behind the ear as treatment method for SHL is favored by some physicians. Li and other authors confirmed that injecting glucocorticoids behind the ear in guinea pigs has longer signal intensity peak duration, longer elimination half-life, longer average residence time, and larger area under the signal time curve.^[20]^ The effectiveness of using postauricular injection to treat sudden hearing loss has been verified by many experts worldwide.^[5,13,15,21,22,23]^ Combining with the glucocorticoid receptors in the inner ear, thereby inhibiting the release of inflammatory response mediators of the Cortis organ and inner ear capillaries, reducing the inflammatory response or improving the immune response state of the inner ear, while reducing the permeability of the inner ear capillaries and stabilizing lysosomes Membrane is the pharmacological mechanism of glucocorticoids in the treatment of sudden hearing loss.^[11,12,24]^ Postauricular injection of glucocorticoids can avoid the blood-labyrinth barrier (BLB) and directly allow the drug to enter the inner ear, increasing the concentration of the drug in the endolymph of the cochlea, which has good drug targeting and purpose.^[25]^ On the other hand, it can reduce systemic adverse reactions, and bring hope to patients with sudden hearing loss accompanied by diabetes, gastric ulcer or other patients who cannot tolerate systemic glucocorticoids.

At present, there are many reports that postauricular injection of glucocorticoid can effectively treat hearing loss, but it is not clear whether this therapy is better than glucocorticoid used in other ways, and what the adverse reactions are. It is necessary to systematically evaluate and meta-analyze the efficacy and adverse effects of glucocorticoid in the treatment of SHL. Although only searching the Chinese and English RCT literature studies may cause selection bias, the specific types of glucocorticoids will affect the results. We will try our best to collect all the data and hope to give an objective evaluation of glucocorticoids used best way to treat SHL.

## Author contributions

All authors have read and approved the final version of the manuscript.

**Conceptualization:** Jiao Liang, Hui Xie, Zhiqing Liu.

**Data analysis:** Jiongke Li, Sha Li.

**Data curation:** Han-Jen Chiang, Sha Li, Jiongke Li, Chenyi Zeng.

**Data collection**: Sha Li, Han-Jen Chiang, Chenyi Zeng.

**Funding acquisition:** Hui Xie.

**Methodology:** Sha Li.

**Study design:** Jiao Liang, Hui Xie, Zhi-qing Liu.

**Supervision:** Hui Xie.

**Writing – review & editing:** Jiao Liang, Han-Jen Chiang.
